# Cytotoxicity screening of Bangladeshi medicinal plant extracts on pancreatic cancer cells

**DOI:** 10.1186/1472-6882-10-52

**Published:** 2010-09-17

**Authors:** Sherine George, Siddharth V Bhalerao, Erich A Lidstone, Irfan S Ahmad, Atiya Abbasi, Brian T Cunningham, Kenneth L Watkin

**Affiliations:** 1 Department of Bioengineering, University of Illinois at Urbana Champaign, USA; 2 Department of Electrical and Computer Engineering, University of Illinois at Urbana Champaign, USA; 3 Center for Nanoscale Science and Technology, Micro and Nanotechnology Laboratory, University of Illinois at Urbana Champaign, USA; 4 Agricultural and Biological Engineering, University of Illinois at Urbana Champaign, USA; 5 International Center for Chemical and Biological Sciences, University of Karachi, Pakistan; 6 Beckman Institute for Advanced Science and Technology, Bio-Imaging Science and Technology Group, University of Illinois at Urbana Champaign, USA

## Abstract

**Background:**

There has been a long standing interest in the identification of medicinal plants and derived natural products for developing cancer therapeutics. Our study focuses upon pancreatic cancer, due to its high mortality rate, that is attributed in part to the lack of an effective chemotherapeutic agent. Previous reports on the use of medicinal plant extracts either alone or alongside conventional anticancer agents in the treatment of this cancer have shown promising results. This work aims to investigate the therapeutic properties of a library of medicinal plants from Bangladesh.

**Methods:**

56 extracts of 44 unique medicinal plants were studied. The extracts were screened for cytotoxicity against the pancreatic adenocarcinoma cell line Panc-1, using a label-free biosensor assay. The top cytotoxic extracts identified in this screen were tested on two additional pancreatic cancer cell lines (Mia-Paca2 and Capan-1) and a fibroblast cell line (Hs68) using an MTT proliferation assay. Finally, one of the most promising extracts was studied using a caspase-3 colorimetric assay to identify induction of apoptosis.

**Results:**

Crude extracts of *Petunia punctata, Alternanthera sessilis*, and *Amoora chittagonga *showed cytotoxicity to three cancer cell lines with IC_50 _values ranging between 20.3 - 31.4 μg/mL, 13.08 - 34.9 μg/mL, and 42.8 - 49.8 μg/mL, respectively. Furthermore, treatment of Panc-1 cells with *Petunia punctata *was shown to increase caspase-3 activity, indicating that the observed cytotoxicity was mediated via apoptosis. Only *Amoora chittagonga *showed low cytotoxicity to fibroblast cells with an IC_50 _value > 100 μg/mL.

**Conclusion:**

Based upon the initial screening work reported here, further studies aimed at the identification of active components of these three extracts and the elucidation of their mechanisms as cancer therapeutics are warranted.

## Background

Pancreatic cancer is the fourth leading cause of cancer-related death in both sexes in the United States [1]. Although Gemcitabine is the current first-line chemotherapeutic administered for metastatic pancreatic cancer, this line of treatment has been met with limited survival and symptomatic outcomes [2,3] resulting in research interest in exploring new alternatives for treatment and prevention. Natural products play a dominant role in the discovery of such new drugs, as over 60% of approved drugs or those in late stages of development (during 1989-1995) are of natural origin [4]. Examples of clinically useful antitumor agents derived from plants include paclitaxel, vincristine, and camptothecin. Many of these plant-derived anticancer agents have been discovered through large-scale screening programs [5]. Furthermore, the broad reaching support and continuation of studies of plant extracts with implications in pancreatic cancer treatment are indicative of the continued role that natural products play in the drug discovery process [6,7].

This study provides data on the cytotoxic potential of 56 extracts derived from 44 different plants used in Bangladeshi folk medicine. A three-tiered screening system was designed, in which all extracts were first screened for their ability to induce death in the Panc-1 cell line using a label-free photonic crystal (PC) biosensor assay. These experiments generated biosensor images of attached cells which were used to quantify cell proliferation changes in treated versus untreated cultures. Next, extracts that showed significant cytotoxicity to Panc-1 cells (> 80% cell death at a testing concentration of 100 μg/mL) in the PC biosensor assay were tested using a colorimetric MTT assay on two additional pancreatic cell lines (Mia-Paca2, and Capan-1). Toxicity to a normal foreskin Hs68 fibroblast cell line was studied as a control. Finally, the extract showing the highest cytotoxicity in all three cancer cell lines was evaluated for its apoptotic activity via a caspase-3 quantification assay.

## Methods

### Plant materials

Fifty-six plant extracts (Table 1) commonly used in Bangladeshi folk medicine were kindly provided by Dr. R. Chowdhury from the University of Dhaka, Bangladesh, where voucher specimens are maintained. The plants were collected from the Dhaka, Chittagonga, and Khulna districts of Bangladesh.

**Table 1:** The names of the 56 plant extracts screened in this study.

Extract No.	Plant	Family Name	Extract/partitionate	Fraction of Panc-1 cell survival
**1**	***Aglaia roxburghiana *Kurz**	**Meliaceae**	**n-Hexane partitionate of MeOH extract**	**0.02 ± 0.00**
2	*Alternanthera sessilis *DC.	Amaranthaceae	Pet-ether fraction of MeOH extract	0.43 ± 0.14
3	*Alternanthera sessilis *DC.	Amaranthaceae	MeOH residue of MeOH extract	1.63 ± 0.41
**4**	***Alternanthera sessilis *DC**.	**Amaranthaceae**	**CHCl3 fraction of MeOH extract**	**0.04 ± 0.09**
5	*Amoora chittagonga *Hiern	Meliaceae	Pet-ether partitionate of MeOH extract	0.36 ± 0.17
**6**	***Amoora chittagonga *Hiern**	**Meliaceae**	**CHCl3 partitionate of MeOH extract**	**0.02 ± 0.01**
7	*Amoora chittagonga *Hiern	Meliaceae	EtOAc partitionate of MeOH extract	2.38 ± 0.35
9	*Amoora rohituka *(Roxb.) Wight & Arn.	Meliaceae	Pet-ether extract	0.10 ± 0.02
10	*Amoora rohituka *(Roxb.) Wight & Arn.	Meliaceae	MeOH extract	0.79 ± 0.18
11	*Anisoptera glabra *Kurz	Dipterocarpaceae	MeOH extract	3.18 ± 0.18
12	*Anogeissus latifolia *(Roxb.) Bedd	Combretaceae	EtOH extract	0.18 ± 0.06
13	*Brunfelsia americana *L.	Solanaceae	MeOH extract	0.79 ± 0.14
14	*Brunfelsia latifolia *Hort. ex. Steud.	Solanaceae	MeOH extract	1.46 ± 0.27
15	*Buchanania lanzen *Spreng.	Anacardiaceae	MeOH extract	0.23 ± 0.11
**16**	***Bursera serrata *Wall. ex. Coleb**.	**Burseraceae**	**Pet-ether extract**	**0.01 ± 0.01**
**17**	***Bursera serrata *Wall. ex. Coleb**.	**Burseraceae**	**Dichloromethane extract**	**0.07 ± 0.03**
18	*Chukrasia tabularis *Juss.	Meliaceae	MeOH extract	0.21 ± 0.11
19	*Cinnamomum zeylanicum *Blume	Lauraceae	MeOH extract	0.59 ± 0.15
**20**	***Citrus hystrix *DC**.	**Rutaceae**	**MeOH extract**	**0.02 ± 0.01**
21	*Combretum coccineum *Engl. & Diels	Combretaceae	CHCl3 partitionate of acidified MeOH extract	0.35 ± 0.12
22	*Combretum grandiflorum *G.Don	Combretaceae	MeOH extract	1.15 ± 0.23
23	*Eclipta prostrata *L.	Asteraceae	MeOH extract	0.50 ± 0.03
24	*Erioglossum edule *Blume	Sapindaceae	MeOH extract	0.93 ± 0.03
25	*Ficus indica *L.	Moraceae	MeOH extract	0.57 ± 0.08
26	*Garuga pinnata *Roxb.	Burseraceae	MeOH extract	0.60 ± 0.19
27	*Indigofera tinctoria *L.	Papilionaceae	EtOH extract	0.77 ± 0.26
28	*Lannea coromandelica *(Houtt.) Merr.	Anacardiaceae	MeOH extract	2.44 ± 0.15
29	*Nephelium litchi *Sonn.	Sapindaceae	MeOH extract	0.27 ± 0.11
30	*Nephelium longan *(Lour.) Hook	Sapindaceae	MeOH extract	0.75 ± 0.19
31	*Pesprum nocturnum *L.	Solanaceae	MeOH extract	0.10 ± 0.02
32	*Petunia meleagris *Planch.	Solanaceae	MeOH extract	1.26 ± 0.29
33	*Petunia phoenicea *H.Jacq.	Solanaceae	MeOH extract	0.66 ± 0.22
**34**	***Petunia punctata *Paxton**	**Solanaceae**	**MeOH extract**	**0.08 ± 0.04**
35	*Petunia violaceae *Lindl.	Solanaceae	MeOH extract	0.46 ± 0.16
**37**	***Phyllanthus reticulatus *Lodd**.	**Euphorbiaceae**	**CHCl3 fraction of pet-ether extract**	**0.03 ± 0.01**
38	*Phyllanthus reticulatus *Lodd.	Euphorbiaceae	Hexane fraction of CH2Cl2 extract	0.60 ± 0.26
39	*Phyllanthus reticulatus *Lodd.	Euphorbiaceae	Acidified CHCl3 fraction of aqueous extract	1.25 ± 0.31
40	*Phyllanthus reticulatus *Lodd.	Euphorbiaceae	Acetone extract	1.36 ± 0.21
**41**	***Poivrea coccinea *DC**.	**Combretaceae**	**MeOH extract**	**0.04 ± 0.01**
42	*Pongamia glabra *Vent.	Leguminosea	MeOH extract	0.14 ± 0.07
43	*Protium serratum *Engl.	Burseraceae	MeOH extract	1.65 ± 0.12
44	*Pterospermum suberifolium *Willd.	Sterculiaceae	MeOH extract	0.59 ± 0.06
45	*Quisqualis indica *L.	Combretaceae	Pet-ether extract	1.19 ± 0.31
46	*Quisqualis indica *L.	Combretaceae	MeOH extract	1.07 ± 0.24
47	*Sapindus mukorossi *Gaertn.	Sapindaceae	MeOH extract	1.57 ± 0.18
48	*Semecarpus anacardium *L.f.	Anacardiaceae	MeOH extract	2.10 ± 0.05
49	*Shorea robusta *C.F.Gaertn.	Dipterocarpaceae	EtOH extract	0.48 ± 0.16
52	*Spondias mangifera *Willd.	Anacardiaceae	MeOH extract	1.83 ± 0.14
53	*Swintonia floribunda *Griff.	Anacardiaceae	MeOH extract	2.92 ± 0.17
54	*Terminalia bellerica *Roxb.	Combretaceae	MeOH extract	0.22 ± 0.05
55	*Terminalia bellerica *Roxb.	Combretaceae	MeOH extract	1.64 ± 0.26
56	*Trachyspermum ammi *Sprague	Umbelliferae	EtOH extract	1.04 ± 0.18
57	*Xanthoxylum budrunga *DC.	Rutaceae	MeOH residue of MeOH extract	0.55 ± 0.05
59	*Xylocurpus molucensis *(Lamarck) M. Roemer	Meliaceae	n-Hexane partitionate of MeOH extract	1.12 ± 0.05
60	*Xylocurpus molucensis *(Lamarck) M. Roemer	Meliaceae	MeOH residue of MeOH extract	0.62 ± 0.18
61	*Zizyphus jujuba *Mill.	Rhamnaceae	MeOH extract	0.90 ± 0.19

The last column presents the anti-proliferative activity of each extract and the top nine cytotoxic extracts are in bold*.

*The PC biosensor was used to screen this library for extracts with anti-proliferative properties and the results are presented as the fraction of Panc-1 (human pancreatic cancer) cell survival relative to untreated controls after exposure to the plant extract. A testing concentration of 100 μg/mL and exposure time of 24 hours was used. Mean ± S.D. values of three replicates are reported here.

### Extraction of plant materials

Details of the extraction process have been described previously [8]. Briefly, the air-dried and powdered leaves of each plant were extracted with light petroleum ether, dichloromethane, ethanol, or methanol. The extraction method used for each sample that was tested is listed in Table 1. The extracts were then filtered and the volume of the filtrate was reduced using a Buchii rotary evaporator at low temperature and pressure. Preliminary phytochemical screening results and the reported major constituents of these extracts have been reported [8].

Stock solutions of the extractives were prepared by dissolution in ethanol. The final concentrations of the extract dilutions in culture were 0.1, 1, 10, and 100 μg/mL. The concentration of ethanol in these dilutions was restricted to no more than 0.5% (v/v) to minimize potential effects of the solvent on cell growth. Doxorubicin hydrochloride (DOX), Curcumin (Cur.), and Staurosporine, used as positive controls, were purchased from Sigma Aldrich (St. Louis, MO, USA).

### General cell culture methods

Three human pancreatic carcinoma cell lines and were used in this study: Panc-1, derived from a mostly differentiated carcinoma [9]; MIA PaCa-2 (MIA), derived from an undifferentiated tumor in the pancreas [10]; and Capan-1, derived from liver metastases of a well-differentiated tumor [11]. The human foreskin fibroblast cell line Hs68 was used as a control. All four cell lines were obtained from ATCC (Rockville, MD, USA). Cell lines were

grown at 37°C and 5% CO_2 _in sterile DMEM medium with 10% fetal bovine serum (Panc-1 and Hs68), DMEM with 10% fetal bovine serum and 2.5% horse serum (MIA), and IMDM with 20% fetal bovine serum (Capan-1), after the addition of 4 mM L-glutamine and penicillin-streptomycin. Cell culture media was obtained from the School of Chemical Sciences Cell Media Facility at the University of Illinois at Urbana-Champaign. Cells were grown in standard tissue culture flasks and upon reaching 80% confluence were passaged with a solution of 0.25% trypsin-EDTA.

### Cytotoxicity screening using PC biosensor-based cell attachment assay

The preliminary determination of the effect of each plant extract on Panc-1 cells was made in an initial screen using a label-free PC biosensor-based assay. Details of the PC biosensor and imaging instrumentation have been described [12,13]. Briefly, the sensor operates by measuring local changes in the peak wavelength value (PWV) of reflected light as cellular binding events occur in the evanescent field that extends above the sensor surface. Sheets of the completed sensor are attached to bottomless 384-well plates. Measurements of the PWV shifts in a microplate well are made on a pixel-by-pixel basis to generate PWV images of the whole well with a spatial resolution of 6.4 μm × 6.4 μm. To measure a shift in the PWV due to cell attachment, the sensor surface is scanned twice: before (baseline scan) and after cells have attached to the surface (post-attachment scan). Using the accompanying software, the two images are aligned and the baseline scan is mathematically subtracted to determine the shift in PWV values due to cell attachment. This PWV shift is a measure of the density of cell binding as a function of position within the well. This method has been used both to detect the attachment of individual cells, and to scan large populations of cells in microplate wells [14,15]. We have also used this assay for cytotoxicity screens, demonstrating excellent agreement in results between the PC and MTT assays [16].

To perform the PC biosensor screening assay, an initial baseline PWV scan was first taken in which each biosensor microplate well contained 25 μL cell culture media. Following this baseline scan, the media was aspirated and the cells were then plated into the wells of a 384-well biosensor microplate at a density of approximately 500 cells/well and allowed to attach overnight at 37°C. To verify cell plating uniformity, a post-attachment scan was taken after cell attachment and the PWV shift image was determined by subtracting the baseline image from the post-attachment image. The cells were then incubated for 24 hours with 25 μL of extract in media at a final concentration of 100 μg/mL. Control cultures and blank wells without cells received 100 μL of medium with 0.5% (v/v) of ethanol. After the 24 h drug exposure period, the cells were grown for an additional 24 h in extract-free fresh medium. Finally, a post-treatment scan was taken and the PWV shift image was determined by subtracting the baseline image from the post-treatment image. Increases in the local PWV as registered by the local pixels were observed only in regions where cells formed a close physical attachment to the sensor surface. Dead cells and other debris that are physically deposited on the same surface did not register similar increases in the local PWV. Estimates of the number of the cells that were attached to the sensor surface in each well were made using a PWV shift threshold as described earlier [17]. Cell counts were determined for all wells after incubation with the extracts and proliferation was expressed as the fraction of treated cells that survived relative to untreated cultures. Every experiment included a set of negative controls (untreated cultures) as well as two sets of positive controls (DOX and Curcumin). All experiments were performed in triplicate.

### MTT proliferation assay

The MTT assay was used to confirm the results of the initial PC-based screen and test identified leads on three additional cell lines. Changes in proliferation rates of cells treated with various plant extracts were determined using the ATCC MTT Cell Proliferation Assay kit. The assay, previously described [18], is based on the conversion of yellow tetrazolium salt MTT to purple formazan crystals by metabolically active cells. The amount of formazan produced is proportional to the number of viable cells.

Cells were plated in 96-well flat bottom tissue culture plates at a density of approximately 1-1.2 × 10^4 ^cells/well and allowed to attach overnight at 37°C. The cells were then incubated with 100 μL of an extract at a concentration of 100 μg/mL for 24 hours. Untreated cultures and blank wells without cells received 100 μL of medium with 0.5% (v/v) of ethanol. After the drug exposure period, the cells were grown for an additional 24 hours in extract-free fresh medium. Next, 10 μL of the MTT reagent was added to each well, and the plate was incubated for 4 hours at 37°C. The MTT crystals were then solubilized overnight by adding 100 μL of the MTT detergent reagent to each well. Absorbance measurements were made at 570 nm using a Biotek^® ^Synergy HT Spectrophotometer. Proliferation was expressed as the fraction of treated cells that survived relative to untreated cultures. The IC_50 _values were calculated using GraphPad Prism 5.0 (GraphPad Software Inc., San Diego, CA). Every experiment included a set of negative controls (untreated cultures) as well as two sets of positive controls (DOX and Curcumin). All experiments were performed in triplicate and repeated at least twice.

### Caspase-3 activity quantification assay

Caspase-3 activity was assessed using the caspase-3 Colorimetric Assay Kit (Sigma Aldrich), following the manufacturer's instructions. This assay is based on the detection of the amount of Ac-DEVD-*p*-NA substrate cleaved by cell lysates to release the colored *p*-NA molecule. Here, PANC-1 cells were exposed to the *P. punctata *extract (100 μg/mL) or Staurosporine (0.1 μg/mL), a known apoptotic agent. Following treatment, the cells were washed in PBS and suspended in a lysis buffer (50 mM HEPES pH 7.4, 5 mM CHAPS, 5 mM DTT) for 15 minutes at a concentration of 10^7 ^cells per 100 μL of buffer. Lysed cells were centrifuged at 16,000 × g, 4°C for 15 minutes. Lysate protein concentrations were determined using the Bradford assay. Equal amounts of protein (~ 20 μg) from each sample were added to wells containing the assay buffer (20 mM HEPES, pH 7.4, 0.1% CHAPS, 5 mM DTT, 2 mM EDTA), followed by 10 μl of Ac-DEVD-*p*-NA (20 mM), bringing the total volume of each well to 100 μl. Caspase-3 activity was assessed by measuring the optical density at 405 nm using a Biotek^® ^Synergy HT Spectrophotometer. The amount of Ac-DEVD-pNA cleaved was then calculated and plotted. The effect of Ac-DEVD-CHO, a caspase-3 inhibitor, on caspase-3 activity was studied simultaneously. As per the manufacturer's instructions, 10 μl of the inhibitor Ac-DEVD-CHO (2 mM) was added to a well containing the assay buffer, followed immediately by the cell lysate and 10 μl of the substrate Ac-DEVD-pNA (20 mM). All experiments were performed in duplicate and repeated at least twice.

### Statistical analysis

Results were expressed as means ± SD of replicates. Comparison between data sets was performed using one way analysis of variance (ANOVA) followed by Student's *t*-test. All statistical analyses were performed using GraphPad Prism 5.0 (GraphPad Software Inc., San Diego, CA). Differences were accepted as statistically significant at *p *< 0.05.

## Results

### Cytotoxicity screening on Panc-1 cells

**Figure 1:**
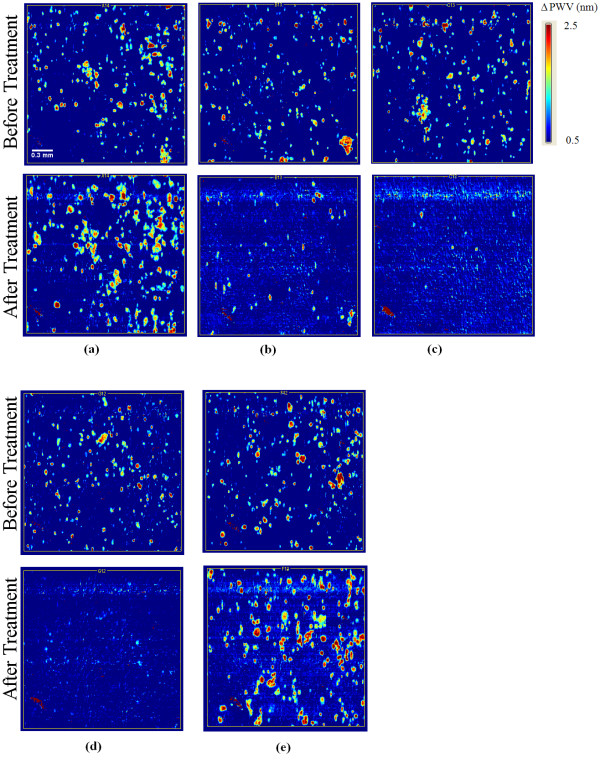
**PWV shift images in false color with shift scale bars indicating the magnitude of wavelength shifts in nanometers**. Pixels with higher PWV displayed in brighter colors, indicate locations where Panc-1 cell attachment has occurred. The five image sets represent the following (a) untreated control, (b) positive control (DOX), (c) positive control (Curcumin), (d) extract that induced 100% cell death (*P. punctata*, No. 34), (e) extract that enhanced proliferation (*A. glabra*, No. 11). In each image set, the top image was taken before exposure and the bottom image was taken after the 24 hour exposure period with a plant extract or positive control at 100 μg/mL. Scale bar (white) = 300 μm.

**Figure 2:**
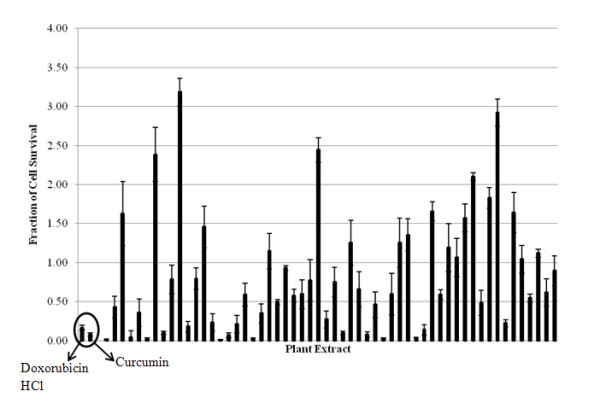
**56 plant extracts were screened for their ability to induce Panc-1 cell death using the PC biosensor assay to determine the fractions of cell surviving after 24 h exposure to the extract**. Dox and Curcumin are the positive controls. Nine extracts induced > 80% cell death. Data represents mean ± S.D. values of three replicates.

An initial screen of fifty-six extracts was performed on the Panc-1 cell line using the PC biosensor-based cell attachment assay. Biosensor images of representative individual wells of a 384-well biosensor microplate before and after treatment are shown in Figure 1. Here, the PWV shift as a function of position is represented as a false color image in which each pixel represents a 6.4 μm × 6.4 μm region of the sensor surface. Figure 1a shows the PWV shift images of an untreated control well taken before and after the treatment period. Figures 1b-c show PWV shift images of the two positive control wells, DOX and Curcumin, taken before and after the treatment period. Figure 1d shows PWV shift images of cells treated with *P. punctata *(No. 34) where nearly 100% cell death was observed. Finally, Figure 1e shows PWV shift images of cells treated with *A. glabra *(No. 11) where the extract enhanced cell proliferation. Using the thresholding method, the number of cells in each well after the initial seeding period was determined from the top PWV shift image. After the treatment period, cell counts for each well were obtained from the bottom PWV shift image and counts from treated wells were compared against those from untreated control wells present on the same plate. The data from PWV shift images of the 56 extracts were converted into cell survival fractions relative to untreated wells as shown in Figure 2. Twenty-two of the plant extracts induced over 50% death of Panc-1 cells at a treatment concentration of 100 μg/mL (Table 1). The top nine cytotoxic extracts induced over 80% cell death.

### Cytotoxicity assay on two additional cancer cell lines and one fibroblast cell line

**Figure 3:**
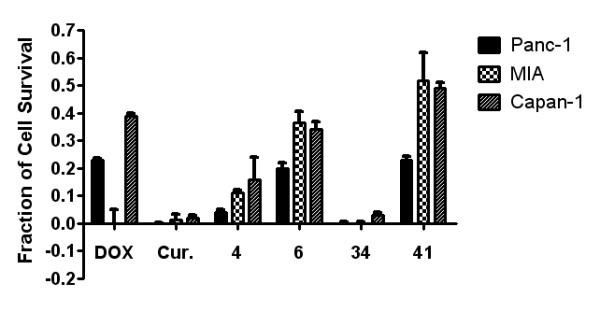
**Subsequent MTT cell viability tests on two additional pancreatic cancer cell lines revealed that four plant extracts (Nos. 4, 6, 34, and 41) were able to suppress proliferation in all three strains**. Reported mean ± S.D. values are from a representative trial out of two or more trials.

**Figure 4:**
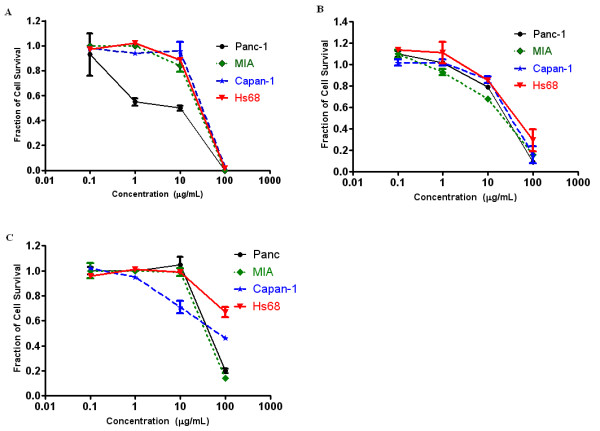
**Growth inhibitory effects of (A) *Petunia punctata *(No. 34), (B) *Althernanthera sessilis *(No. 4), and (C) *Amoora chittagonga *(No. 6) on the three pancreatic cancer cell lines, Panc-1, MIA, and Capan-1, and a human fibroblast cell line, Hs68**. Proliferation suppression appears to be dose dependant. Reported mean ± S.D. values are from a representative trial out of two or more trials.

The nine extracts identified in the PC-based screen were then tested using the MTT assay on the Capan-1, and MIA cell lines, in addition to the Panc-1 cells. Four of these extracts (Nos. 4, 6, 34, 41) induced over 50% death in the MIA and Capan-1 cell lines (Figure 3). The three most potent extracts identified - *P. punctata *(No. 34), *A. sessilis *(No. 4), and *A. chittagonga *(No. 6) - were found to induce over 70% cell death in all three cell lines. Dose response studies of these three extracts on three cancer cell lines and one fibroblast cell line (Hs68) are presented in Figure 4 and IC_50 _values are summarized in Table 2. The *P. punctata *extract had IC_50 _values of 20.34 μg/mL, 24.97 μg/mL, and 31.39 μg/mL for Panc-1, MIA, and Capan-1, respectively. However, this extract was also toxic to Hs68 cells with an IC_50 _value of 25.81 μg/mL. Similarly, the *A. sessilis *(No. 4) extract showed high cytotoxicity to the cancer cell lines with IC_50 _values in the 13.08 μg/mL - 34.92 μg/mL range but limited selectivity. In contrast, *A. chittagonga *(No.6) showed high cytotoxicity to all cancer cells (IC_50 _of 42.8 μg/mL - 49.8 μg/mL) and low cytotoxicity to the fibroblast cells (IC_50 _> 100 μg/mL)

**Table 2:** IC_50 _values of *P. punctata *(Extract No. 34), *A. sessilis *(No. 4) and *A. chittagonga *(No. 6) for human pancreatic cancer and human foreskin fibroblast cell lines.

Cell line	IC_50 _(μg/mL)		
	
	*P. punctata *(No. 34)	*A. sessilis *(No. 4)	*A. chittagonga *(No. 6)
Panc-1	20.34 ± 13.99	27.19 ± 3.01	48.60 ± 4.59
MIA	24.97 ± 4.59	13.08 ± 10.40	42.79 ± 5.88
Capan-1	31.39 ± 2.91	34.92 ± 2.20	49.82 ± 11.60
			
Hs68	25.81 ± 0.44	32.82 ± 10.74	> 100

Mean ± S.D. values of two or more independent trials are reported below.

### Extract induces apoptosis

**Figure 5:**
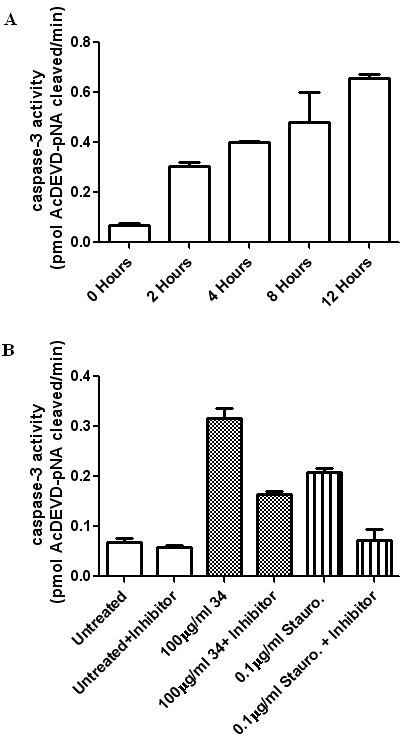
**(A) Caspase-3 activity of Panc-1 cells treated with 100 μg/ml of *Petunia punctata *(No. 34)**. (B) Caspase-3 activity of lysates from untreated, 34-treated, and Staurosporine treated Panc-1 cells in the absence and presence of a caspase-3 inhibitor (Ac-DEVD-CHO). Treatment period lasted three hours.

Caspase-3 activation is a crucial component in the apoptotic signaling cascade. Although *P. punctata *(No. 34) was not selectively cytotoxic to pancreatic cancer cells, we were interested to see if the observed high cytotoxicity to Panc-1 cells treated with *P. punctata *was mediated via apoptosis. To further elucidate the mechanism of cell death induced by *P. punctata*, a caspase-3 colorimetric assay was conducted to establish the levels of caspase-3 activation both before and after treatment with the extract. The results of this experiment show that treatment of Panc-1 cells with *P. punctata *strongly induces increased caspase-3 activity (Figure 5a).

Next, we were interested in comparing the level of *P. punctata*-induced caspase-3 activity to that induced by one of our positive controls. Staurosporine was chosen as an appropriate positive control. It is of note that, at a testing concentration of 100 ug/mL and exposure time of 24 hours, both Staurosprine (fraction of cell survival = 0.07 ± 0.02) and Curcumin exhibited the same level of cytotoxicity on Panc-1 cells as measured through an MTT assay. Figure 5b shows that the caspase-3 activity observed in cells treated with *P. punctata *was similar to that observed with Staurosporine-treated cells. In contrast, untreated controls were shown to maintain a basal caspase-3 activity (Figure 5b). Furthermore, caspase-3 activity in *P. punctata*-treated cells was reduced in the presence of a caspase-3 specific apoptosis inhibitor, Ac-DEVD-CHO. This suggests the involvement of caspase-3 in triggering apoptosis in *P. punctata*-treated Panc-1 cells. Complete inhibition of caspase-3 activity was not observed; this was to be expected, given the brevity of the inhibitor-lysate incubation and the presence of the substrate in 10-fold excess.

## Discussion

Due to the high mortality rates of pancreatic cancer and the absence of effective chemotherapy, there is a continued need for new alternatives for treatment and prevention, and natural products play a dominant role in the discovery of such new drugs [7,19]. In this report, we screened a library of plants native to Bangladesh, many of which are used in Bangladeshi folk medicine for treating a variety of ailments, including cancer. We have used a label-free biosensor assay in conjunction with traditional colorimetric proliferation and caspase-3 activity assays, for identifying new natural-product based leads with high cytotoxicity to pancreatic cancer cells. Three human pancreatic adenocarcinoma cell lines (Panc-1, MIA, and Capan-1) were studied as an *in vitro *model due to both their reported low sensitivity to a current treatment agent (Gemcitabine) and their expression of multiple drug resistant proteins [20-23]. Our experimental results confirmed the drug resistant properties of these cells; Gemcitabine exhibited no cytotoxicity toward Panc-1 cells at a testing concentration of 100 μg/mL (data not shown). Hence, we did not use Gemcitabine as a positive control in our screen.

At a testing concentration of 100 μg/mL, 22 of the 56 extracts reduced Panc-1 cell survival by more than 50% (Table 1 and Figure 2). Upon expanding testing to a panel of two additional pancreatic cancer cell lines and one fibroblast cell line, the following three extracts were found to significantly reduce pancreatic cancer cell proliferation: *P. punctata *(No. 34), *A. sessilis *(No. 4), and *A. chittagonga *(No. 6). While all three extracts showed low cytotoxicity to fibroblast cells at low concentrations (< 20 μg/mL), only *A. chittagonga *continued to exhibit low cytotoxicity at elevated concentrations (Figures 3 and 4).

*P. punctata *was found to show high cytotoxicity against the cancer cell panel. Morphological changes observed in treated cells suggest the induction of cellular apoptosis, which was confirmed by a biochemical assay verifying increased expression of caspase-3 protein in cells treated with *P. punctata *(Figure 5). Interestingly, this study is not the first instance in which the species *P. punctata *(a member of the Solanaceae family) has been associated with potential pharmacological activity. The results of a phytochemical screening of the methanolic extract of *P. punctata *showed the presence of steroids as an active component [8]. Moreover, several additional species of the Solanaceae family are currently used for the treatment of infections and skin disorders [24]. *P. punctata *has also been shown to possess strong antimicrobial activity [25]. In addition, separate studies have reported the antiproliferative activity of species from the Solanaceae family on several human cancer cell lines [26,27].

Similar to *P. punctata*, *A. sessilis *has shown promise in relatively disparate areas of medicine. A member of the Amaranthaceae family, *A. sessilis *has a history of use in Indian and Chinese traditional medicine [27]. Preparations of this plant have been shown to possess anti-microbial properties, in addition to speeding the wound-healing process [27]. A recent study has also demonstrated the hepatoprotective effects of this plant in a rat model [28,29]. However, this represents the first instance in which the therapeutic potential of *A. sessilis *in cancer treatment has been reported.

Finally, *Amoora chittagonga *belongs to the Meliaceae family. A few *Amoora *species have been shown to possess anti-inflammatory, anti-cancer, and hepatoprotective medicinal properties [30]. The compound amooranin, extracted from the bark of an extensively studied Amoora species (*A. rohituka*) has been shown to induce apoptosis in breast carcinoma through caspase activation [31]. Furthermore, this compound is known to be effective against a broad panel of cancer cell lines, including colon cancer, cervical cancer, and leukemia [32]. However, there are currently no reports on the medicinal properties of the *A. chittagonga*. We found that *A. chittagonga *showed anti-proliferative properties in several models of pancreatic cancer, while maintaining low toxicity to non-cancerous Hs68 fibroblast cells.

While not the primary focus of this work, it is worthwhile to discuss some of the advantages afforded by the PC biosensor system in our cell-based screening assays. Since these biosensors are incorporated into microplates, they can be used with standard microplate liquid handling instrumentation such as pipetting systems and wash stations. Furthermore, assays using as few as 500 Panc-1 cells per well in a 384-well plate can be performed, which is particularly important for cell lines that proliferate slowly. A comparable colorimetric based MTT proliferation assay requires 2500-3000 cells per well. Although the assays shown in this work utilize cells that readily attach to the biosensor surface without the use of specific capture molecules, previous work has also demonstrated the use of PC biosensors for cells that would ordinarily remain suspended in solution by preparing the biosensor with immobilized proteins that specifically bind with outer membrane proteins [14]. We have previously demonstrated high correlation between results from the PC-based cell assay and the comparable MTT assay [16].

Since PC biosensor assays are label-free, cells are not stained, killed, or altered in any way. Hence, it is possible to use the biosensor to perform proliferation assays, and to subsequently use the same cells to interrogate the presence of a protein, such as caspase-3. Caspase-3 protein detection and quantification can be performed using commercially available kits designed to work directly with cultures of adherent cells. One such test (Caspase-Glo^® ^from Promega, WI, USA) uses a single reagent to perform both the cell lysis and the caspase cleavage of a substrate leading to the generation of luminescence, which is detectable using any standard microplate-compatible spectrophotometer. Such additional interrogation allows much more information to be derived from a single culture of cells, thereby decreasing the role of biological variability on the results of a given trial, experiment, or screening assay.

## Conclusion

In this work, we screened 56 extracts of 44 unique Bangladeshi medicinal plants for their anti-proliferative properties in three cultured pancreatic cancer cell lines. Screening of each of the extracts identified three extracts with the desired properties. Further testing revealed one extract to induce high toxicity to multiple cancerous cell lines while preserving relatively low levels of toxicity toward a non-cancerous control cell line. Our results provide the basis for the further investigation of each of these species and potential identification of novel bioactive compounds with therapeutic, anti-cancer properties. Elucidating the mechanisms by which these anti-cancer properties are derived from the plant extracts is of crucial importance. Such clarification is necessary to identify, select for, and optimize therapeutic compounds. Though naturally occurring compounds such as those found in *P. punctata*, *A. sesselis*, and *A. chittagonga *often provide excellent starting points, deriving the mechanistic advantages of such compounds will allow the development and realization of specific, highly potent anti-cancer drugs with limited peripheral toxicity.

## Competing interests

Brian T. Cunningham discloses that he is a founder of SRU Biosystems, who provides PC biosensors and detection instruments on a commercial basis.

## Authors' contributions

SG performed the PC assays, MTT assays, and analyzed the resulting data. SG, SVB, and EAL performed the caspase-3 assays and analyzed the data. SG, BTC, EAL, and KLW wrote and reviewed the manuscript. ISA, AA, BTC, and KLW are co-investigators of this project. All authors read and approved the final manuscript.

## Pre-publication history

The pre-publication history for this paper can be accessed here:

http://www.biomedcentral.com/1472-6882/10/52/prepub

## References

[B1] Jemal A Siegel R Ward E Hao Y Xu J Murray T Thun MJ Cancer statistics, 2008 CA Cancer J Clin 2008 58 2 71 96 10.3322/CA.2007.0010 18287387

[B2] Burris HA Moore MJ Andersen J Green MR Rothenberg ML Modiano MR Cripps MC Portenoy RK Storniolo AM Tarassoff P Improvements in survival and clinical benefit with gemcitabine as first-line therapy for patients with advanced pancreas cancer: a randomized trial Journal of Clinical Oncology 1997 15 6 2403 2413 9196156 10.1200/JCO.1997.15.6.2403

[B3] Heinemann V Gemcitabine: progress in the treatment of pancreatic cancer Oncology 2001 60 8 18 10.1159/000055290 11150902

[B4] Cragg GM Newman DJ Snader KM Natural Products in Drug Discovery and Development Journal of Natural Products 1997 60 1 52 60 10.1021/np9604893 9014353

[B5] Pezzuto JM Plant-derived anticancer agents Biochemical pharmacology 1997 53 2 121 133 10.1016/S0006-2952(96)00654-5 9037244

[B6] Schwarz RE Donohue CA Sadava D Kane SE Pancreatic cancer in vitro toxicity mediated by Chinese herbs SPES and PC-SPES: implications for monotherapy and combination treatment Cancer Letters 2003 189 1 59 68 10.1016/S0304-3835(02)00501-3 12445678

[B7] Lau ST Lin ZX Liao Y Zhao M Cheng CHK Leung PS Brucein D induces apoptosis in pancreatic adenocarcinoma cell line PANC-1 through the activation of p38-mitogen activated protein kinase Cancer Letters 2009 281 1 42 52 10.1016/j.canlet.2009.02.017 19286308

[B8] Rahman MS Begum B Chowdhury R Rahman KM Rashid MA Preliminary Cytotoxicity Screening of Some Medicinal Plants of Bangladesh Dhaka University Journal of Pharmaceutical Sciences 2008 7 1 47 52

[B9] Lieber M Mazzetta J Nelson-Rees W Kaplan M Todaro G Establishment of a continuous tumor-cell line (panc-1) from a human carcinoma of the exocrine pancreas International Journal of Cancer 1975 15 5 10.1002/ijc.2910150505 1140870

[B10] Yunis AA Arimura GK Russin DJ Human pancreatic carcinoma (MIA PaCa-2) in continuous culture: sensitivity to asparaginase International Journal of Cancer 1977 19 1 10.1002/ijc.2910190118 832918

[B11] Fogh J Wright WC Loveless JD Absence of HeLa cell contamination in 169 cell lines derived from human tumors Journal of the National Cancer Institute 1977 58 2 209 833871 10.1093/jnci/58.2.209

[B12] Cunningham BT Li P Schulz S Lin B Baird C Gerstenmaier J Genick C Wang F Fine E Laing L Label-free assays on the BIND system Journal of Biomolecular Screening 2004 9 6 481 10.1177/1087057104267604 15452334

[B13] Li PY Lin B Gerstenmaier J Cunningham BT A new method for label-free imaging of biomolecular interactions Sensors & Actuators: B Chemical 2004 99 1 6 13

[B14] Lin B Li P Cunningham BT A label-free biosensor-based cell attachment assay for characterization of cell surface molecules Sensors & Actuators: B Chemical 2006 114 2 559 564

[B15] Cunningham BT Laing L Microplate-based, label-free detection of biomolecular interactions: applications in proteomics Expert Rev Proteomics 2006 3 3 271 281 10.1586/14789450.3.3.271 16771700

[B16] Chan LL Gosangari SL Watkin KL Cunningham BT Label-free imaging of cancer cells using photonic crystal biosensors and application to cytotoxicity screening of a natural compound library Sensors & Actuators: B Chemical 2008 132 2 418 425

[B17] Chan LL Gosangari SL Watkin KL Cunningham BT A label-free photonic crystal biosensor imaging method for detection of cancer cell cytotoxicity and proliferation Apoptosis 2007 12 6 1061 1068 10.1007/s10495-006-0031-y 17252197

[B18] Pieters R Huismans DR Leyva A Veerman AJP Adaptation of the rapid automated tetrazolium dye based(MTT) assay for chemosensitivity testing in childhood leukemia Cancer letters 1988 41 3 323 332 10.1016/0304-3835(88)90294-7 3165705

[B19] Lau ST Lin ZX Leung PS Role of reactive oxygen species in brucein D-mediated p38-mitogen-activated protein kinase and nuclear factor- B signalling pathways in human pancreatic adenocarcinoma cells British Journal of Cancer 2010 102 583 593 10.1038/sj.bjc.6605487 20068565 PMC2822930

[B20] Piacentini P Donadelli M Costanzo C Moore PS Palmieri M Scarpa A Trichostatin A enhances the response of chemotherapeutic agents in inhibiting pancreatic cancer cell proliferation Virchows Archiv 2006 448 6 797 804 10.1007/s00428-006-0173-x 16568310

[B21] Miller DW Fontain M Kolar C Lawson T The expression of multidrug resistance-associated protein (MRP) in pancreatic adenocarcinoma cell lines Cancer Letters 1996 107 2 301 10.1016/0304-3835(96)04384-4 8947528

[B22] Giroux V Iovanna J Dagorn JC Probing the human kinome for kinases involved in pancreatic cancer cell survival and gemcitabine resistance The FASEB Journal 2006 20 12 1982 10.1096/fj.06-6239com 17012250

[B23] Lau ST Lin ZX Zhao M Leung PS Brucea javanica fruit induces cytotoxicity and apoptosis in pancreatic adenocarcinoma cell lines Phytotherapy Research 2008 22 4 10.1002/ptr.2344 18386257

[B24] Mongelli E Coussio J Ciccia G Maestri D Zygadlo J Medicinal species of the Solanaceae family: Primary screening of cytotoxicity 1997 ISHS 177 180

[B25] Rahman MS Rahman MZ Wahab MA Chowdhury R Rashid MA Antimicrobial Activity of Some Indigenous Plants of Bangladesh Dhaka University Journal of Pharmaceutical Sciences 2008 7 1 23 26

[B26] Chiang HC Jaw SM Chen CF Kan WS Antitumor agent, physalin F from Physalis angulata L Anticancer research 1992 12 3 837 843 1622143

[B27] Mothana RAA Grunert R Lindequist U Bednarski PJ Study of the anticancer potential of Yemeni plants used in folk medicine Pharmazie 2007 62 4 305 307 17484289

[B28] Jalalpure SA Agrawal N Patil M Chimkode R Tripathi A Antimicrobial and wound healing activities of leaves of Alternanthera sessilis Linn Intl J of Green Pharmacy 2008 2

[B29] Lin SC Lin CC Shyuu SJ Lin YH Hepatoprotective effects of Taiwan folk medicine: Alternanthera sessilis on liver damage induced by various hepatotoxins Phytotherapy Research 1994 8 7 391 398 10.1002/ptr.2650080703

[B30] Rabi T Gupta RC Antitumor and cytotoxic investigation of Amoora rohituka Pharmaceutical Biology 1995 33 4 359 361 10.3109/13880209509065396

[B31] Rabi T Ramachandran C Fonseca HB Nair RPK Alamo A Melnick SJ Escalon E Novel drug amooranin induces apoptosis through caspase activity in human breast carcinoma cell lines Breast cancer research and treatment 2003 80 3 321 330 10.1023/A:1024911925623 14503804

[B32] Ramachandran C Nair PK Alamo A Cochrane CB Escalon E Melnick SJ Anticancer effects of amooranin in human colon carcinoma cell line in vitro and in nude mice xenografts International Journal of Cancer 2006 119 10 2443 2454 10.1002/ijc.22174 16894569

